# Supplementary material for: The impact of working in academia on researchers’ mental health and well-being: A systematic review and qualitative meta-synthesis
Source: PLoS One. 2022 May 25;17(5):e0268890. doi: 10.1371/journal.pone.0268890 (PMC9132292; doi:10.1371/journal.pone.0268890)
Supplement: S2 File — (PDF) [file pone.0268890.s002.pdf]

Search strategy

The SPIDER tool was used to construct a search strategy for each of the following bibliographic databases: PubMed, PsycINFO (Ovid), CINAHL Plus, SCOPUS, Web of Science, and EMBASE (Ovid).

The search syntax is as follows: S AND PI AND (D OR E OR R).

The search terms that were used to search for literature in Google Scholar are also displayed below.

Key:

[ti,ab] – title or abstract

[ti,id,ab] – title or keyword or abstract

[TI,AB] – title or abstract

[TITLE] - title

[TITLE-ABS-KEY] – title or abstract or keyword

[ab,kw,ti] – abstract or keyword or title

Google Scholar search terms

"mental health of researchers"
(well-being OR wellbeing OR "well being") AND researchers
"research environment" AND (university OR academia OR "higher education") AND (stress OR depression OR anxiety)
"university staff" AND (stress OR anxiety OR depression) AND qualitative

	**PubMed**<br>[mh] – MeSh Terms	**PsycINFO (Ovid)**<br>/ - subject heading	**CINAHL Plus**<br>MH – CINAHL subject heading
**S**<br>(Sample)	(research personnel[mh] OR education, graduate[mh]) OR ("researcher*" OR "scholar*" OR "lecturer*" OR "facult*" OR "research staff" OR "PhD student" OR "doctoral student" OR "doctorate*" OR "postdoc*" OR "post docs" OR "post doc" OR "post-doc" OR "post-docs" OR "post-doctoral" OR "post doctoral" OR "research associate*" OR "research fellow*" OR "research assistant*" OR "principal investigator" OR "scientist*" OR "professor*" [ti,ab]).<br><br>AND<br><br>(organizational culture[mh]) OR ("job" OR "jobs" OR "employ*" OR "occupation*" OR "career*" OR "workplace*" OR "work-place*" OR "work place*" OR "organizational culture*" OR "organizational climate*" OR "research culture*" OR "research environment*" [ti,ab]).<br><br>AND<br><br>("universit*" OR "higher education" OR "academia" OR "academic*" OR "academe" [ti,ab]).	(graduate education/) OR (researcher* OR scholar* OR lecturer* OR facult* OR research staff OR PhD student OR doctoral student OR doctorate* OR postdoc* OR post docs OR post doc OR post-doc OR post-docs OR post-doctoral OR post doctoral OR research associate* OR research fellow* OR research assistant* OR principal investigator OR scientist* OR professor* [ti,id,ab]).<br><br>AND<br><br>(organizational climate/) OR (job OR jobs OR employ* OR occupation* OR career* OR workplace* OR work-place* OR work place* OR organizational culture* OR organizational climate* OR research culture* OR research environment* [ti,id,ab])<br><br>AND<br><br>(universit* OR higher education OR academia OR academic* OR academe [ti,id,ab]).	(MH "Research Personnel" OR MH "Education, Graduate") OR ("researcher*" OR "scholar*" OR "lecturer*" OR "facult*" OR "research staff" OR "PhD student" OR "doctoral student" OR "doctorate*" OR "postdoc*" OR "post docs" OR "post doc" OR "post-doc" OR "post-docs" OR "post-doctoral" OR "post doctoral" OR "research associate*" OR "research fellow*" OR "research assistant*" OR "principal investigator" OR "scientist*" OR "professor*" [TI,AB]).<br><br>AND<br><br>MH ''Organizational Culture'') OR ("job" OR "jobs" OR "employ*" OR "occupation*" OR "career*" OR "workplace*" OR "work-place*" OR "work place*" OR "organizational culture*" OR "organizational climate*" OR "research culture*" OR "research environment*" (TI,AB]).<br><br>AND<br><br>("universit*" OR "higher education" OR "academia" OR "academic*" OR "academe" [TI, AB]).
**PI**<br>(Phenomenon of interest)	(mental health[mh] OR burnout, professional[mh] OR adaptation, psychological[mh] OR resilience, psychological[mh] OR personal satisfaction[mh] OR quality of life[mh] OR job satisfaction[mh]) OR ("mental health" OR "mental illness*" OR "disorder*" OR "depress*" OR "low mood" OR "anxiety" OR "anxious" OR "wellbeing" OR "well being" OR "well-being" OR "stress*" OR "distress*" OR "burnout" OR "resilience" OR "adapt*" OR "cope*" OR "coping" OR "quality of life" OR "life satisfaction" OR "personal satisfaction" OR "job satisfaction" [ti,ab]).	(mental health/ OR well being/ OR "quality of life"/ OR job satisfaction/ OR occupational stress/ OR "resilience (psychological)"/ OR adaptation/ OR life satisfaction/) OR (mental health OR mental illness* OR disorder* OR depress* OR low mood OR anxiety OR anxious OR wellbeing OR well-being OR well being OR stress* OR distress* OR burnout OR resilience OR adapt* OR cope* OR coping OR quality of life OR life satisfaction OR personal satisfaction OR job satisfaction [ti,id,ab]).	(MH "Mental Health" OR MH "Stress, Occupational" OR MH "Quality of Life" OR MH "Adaptation, Psychological" OR MH "Job Satisfaction" OR MH "Personal Satisfaction") OR ("mental health" OR "mental illness*" OR "disorder*" OR "depress*" OR "low mood" OR "anxiety" OR "anxious" OR "wellbeing" OR "well-being" OR "well being" OR "stress*" OR "distress*" OR "burnout" OR "resilience" OR "adapt*" OR "cope*" OR "coping" OR "quality of life" OR "life satisfaction" OR "personal satisfaction" OR "job satisfaction" [TI,AB]).
**D**<br>(Design)	("thematic analysis" OR "thematic synthesis" OR "grounded theory" OR "discourse analysis" OR "phenomenolog*" OR "content analysis" OR "interview*" OR "focus group*" OR "observation*" OR "ethnograph*" OR "case study" OR "case studies" OR "lived experience" OR "life experience" OR "story" OR "stories" OR "narrative*" OR "narration*" OR "commentar*" [ti,ab]).	(thematic analysis OR thematic synthesis OR grounded theory OR discourse analysis OR phenomenolog* OR content analysis OR interview* OR focus group* OR observation* OR ethnograph* OR case study OR case studies OR lived experience OR life experience OR story OR stories OR narrative* OR narration* OR commentar* [ti,id,ab]).	("thematic analysis" OR "thematic synthesis" OR "grounded theory" OR "discourse analysis" OR "phenomenolog*" OR "content analysis" OR "interview*" OR "focus group*" OR "observation*" OR "ethnograph*" OR "case study" OR "case studies" OR "lived experience" OR "life experience" OR "story" OR "stories" OR "narrative*" OR "narration*" OR "commentar*" [TI,AB]).
**E**<br>(Evaluation)	("opinion*" OR "perspective*" OR "attitude*" OR "experience*" OR "feel*" OR "belie*" OR "perception*" OR "thought*" OR "view*" OR "expectation*" [ti,ab]).	(opinion* OR perspective* OR attitude* OR experience* OR feel* OR belie* OR perception* OR thought* OR view* OR expectation* [ti,id,ab]).	("opinion*" OR "perspective*" OR "attitude*" OR "experience*" OR "feel*" OR "belie*" OR "perception*" OR "thought*" OR "view*" OR "expectation*" [TI,AB])
**R**<br>(Research type)	("qualitative research"[mh]) OR ("qualitative" [ti,ab]).	(qualitative methods/) OR (qualitative [ti,id,ab]).	(MH "Qualitative Studies") OR ("qualitative" [TI,AB]).

	SCOPUS	Web of Science	EMBASE (Ovid)<br>/ - Emtree subject heading
**S**<br>(Sample)	(“researcher*” OR “scholar*” OR “lecturer*” OR “facult*” OR “research staff” OR “PhD student” OR “doctoral student” OR “doctorate*” OR “postdoc*” OR “post docs” OR “post doc” OR “post-doc” OR “post-docs” OR “post-doctoral” OR “post doctoral” OR “research associate*” OR “research fellow*” OR “research assistant*” OR “principal investigator” OR “scientist*” OR “professor*” [TITLE-ABS-KEY]).<br><br>AND<br><br>(“job” OR “jobs” OR “employ*” OR “occupation*” OR “career*” OR “workplace*” OR “work-place*” OR “work place*” OR “organizational culture*” OR “organizational climate*” OR “research culture*” OR “research environment*” [TITLE-ABS-KEY]).<br><br>AND<br><br>(“universit*” OR “higher education” OR “academic* OR “academia” OR “academe” [TITLE-ABS-KEY]).	(“researcher*” OR “scholar*” OR “lecturer*” OR “facult*” OR “research staff” OR “PhD student” OR “doctoral student” OR “doctorate*” OR “postdoc*” OR “post docs” OR “post doc” OR “post-doc” OR “post-docs” OR “post-doctoral” OR “post doctoral” OR “research associate*” OR “research fellow*” OR “research assistant*” OR “principal investigator” OR “scientist*” OR “professor*” [TI,AB]).<br><br>AND<br><br>(“job” OR “jobs” OR “employ*” OR “occupation*” OR “career*” OR “workplace*” OR “work-place*” OR “work place*” OR “organizational culture*” OR “organizational climate*” OR “research culture*” OR “research environment*” [TI,AB]).<br><br>AND<br><br>(“universit*” OR “higher education” OR “academic* OR “academia” OR “academe” [TI,AB]).	(researcher* OR scholar* OR lecturer* OR facult* OR research staff OR PhD student OR doctoral student OR doctorate* OR postdoc* OR post docs OR post doc OR post-doc OR post-docs OR post-doctoral OR post doctoral OR research associate* OR research fellow* OR research assistant* OR principal investigator OR scientist* OR professor* [ab,kw,ti]).<br><br>AND<br><br>(organizational culture/ OR organizational climate/) OR (job OR jobs OR employ* OR occupation* OR career* OR workplace* OR work-place* OR work place* OR organizational culture* OR organizational climate* OR research culture* OR research environment* [ab,kw,ti]).<br><br>AND<br><br>(universit* OR higher education OR academia OR academic* OR academe [ab,kw,ti]).
**PI**<br>(Phenomenon of interest)	(“mental health” OR “mental illness*” OR “disorder*” OR “depress*” OR “low mood” OR “anxiety” OR “anxious” OR “wellbeing” OR “well being” OR “well-being” OR “stress*” OR “distress*” OR “burnout” OR “resilience” OR “adapt*” OR “cope*” OR “coping” OR “quality of life” OR “life satisfaction” OR “personal satisfaction” OR “job satisfaction” [TITLE-ABS-KEY]).	(“mental health” OR “mental illness*” OR “disorder*” OR “depress*” OR “low mood” OR “anxiety” OR “anxious” OR “wellbeing” OR “well being” OR “well-being” OR “stress*” OR “distress*” OR “burnout” OR “resilience” OR “adapt*” OR “cope*” OR “coping” OR “quality of life” OR “life satisfaction” OR “personal satisfaction” OR “job satisfaction” [TI,AB]).	(mental health/ OR wellbeing/ OR job stress/ OR professional burnout/ OR coping behaviour/ OR job satisfaction/ OR life satisfaction/ OR “quality of life”/) OR (mental health OR mental illness* OR disorder* OR depress* OR low mood OR anxiety OR anxious OR wellbeing OR well-being OR well being OR stress* OR distress* OR burnout OR resilience OR adapt* OR cope* OR coping OR quality of life OR life satisfaction OR personal satisfaction OR job satisfaction [ab,kw,ti]).
**D**<br>(Design)	(“thematic analysis” OR “thematic synthesis” OR “grounded theory” OR “discourse analysis” OR “phenomenolog*” OR “content analysis” OR “interview*” OR “focus group*” OR “observation*” OR “ethnograph*” OR “case study” OR “case studies” OR “lived experience” OR “life experience” OR “story” OR “stories” OR “narrative*” OR “narration*” OR “commentar*” [TITLE-ABS-KEY]).	(“thematic analysis” OR “thematic synthesis” OR “grounded theory” OR “discourse analysis” OR “phenomenolog*” OR “content analysis” OR “interview*” OR “focus group*” OR “observation*” OR “ethnograph*” OR “case study” OR “case studies” OR “lived experience” OR “life experience” OR “story” OR “stories” OR “narrative*” OR “narration*” OR “commentar*” [TI,AB])	(thematic analysis OR thematic synthesis OR grounded theory OR discourse analysis OR phenomenolog* OR content analysis OR interview* OR focus group* OR observation* OR ethnograph* OR case study OR case studies OR lived experience OR life experience OR story OR stories OR narrative* OR narration* OR commentar* [ab,kw,ti]).
**E**<br>(Evaluation)	(“opinion*” OR “perspective*” OR “attitude*” OR “experience*” OR “feel*” OR “belie*” OR “perception*” OR “thought*” OR “view*” OR “expectation*” [TITLE-ABS-KEY]).	(“opinion*” OR “perspective*” OR “attitude*” OR “experience*” OR “feel*” OR “belie*” OR “perception*” OR “thought*” OR “view*” OR “expectation*” [TI,AB]).	(opinion* OR perspective* OR attitude* OR experience* OR feel* OR belie* OR perception* OR thought* OR view* OR expectation* [ab,kw,ti]).
**R**<br>(Research type)	(“qualitative” [TITLE-ABS-KEY]).	(“qualitative” [TI,AB]).	(qualitative research/) OR (qualitative [ab,kw,ti]).
