# Supplementary material for: The impact of working in academia on researchers’ mental health and well-being: A systematic review and qualitative meta-synthesis
Source: PLoS One. 2022 May 25;17(5):e0268890. doi: 10.1371/journal.pone.0268890 (PMC9132292; doi:10.1371/journal.pone.0268890)
Supplement: Attachment — Submitted filename: Response to Reviewers.docx [file pone.0268890.s003.docx]

**UCL Division of Psychiatry**

Helen Nicholls

UCL Division of Psychiatry

149 Tottenham Court Road

London, W1T 7NF, UK

helen.nicholls.20@ucl.ac.uk

Michelle Torok,

Academic Editor,

PLOS ONE

12^th^ April, 2022

Dear Dr Torok,

We would like to thank both you and the reviewers for your valuable and insightful comments on our manuscript entitled: The impact of working in academia on researchers' mental health and well-being: A systematic review and qualitative meta-synthesis (PONE-D-22-03203).

We have provided a point-by-point response to both yours and the reviewers comments in the below table.

| Comments from Academic Editor | | Location of revisions |
| --- | --- | --- |
| This is a well-written, and methodologically sound meta-synthesis of the literature on the relationship between academia and mental health/well-being.  In addition to the reviewer comments noted below, it would be useful for the authors to include a brief overview in the Introduction of what quantitative-focused studies in this area have found. There are many validated mental health and well-being scales which provide a empirical sense of these issues, and for readers unfamiliar with this area, understanding the prevalence and severity of mental ill-health among academics would be useful. The authors could then better highlight the current gaps in understanding about this problem given limitations of quantitative-focused studies as way to strengthen their case for only focusing on qualitative studies. | Thank you for this positive feedback.  We agree that an overview of the quantitative literature would be beneficial. In the introduction, we have included a brief paragraph detailing some of the quantitative literature available on this topic which highlights the prevalence and severity of mental ill health among academic researchers. We have pointed out some of the limitations of this research and linked this to why we have chosen to focus on synthesizing qualitative work in our systematic review. | -  Lines 86-118 |
| Please ensure that your manuscript meets PLOS ONE's style requirements, including those for file naming. | We have carefully checked PLOS ONE’s style requirements and are confident that our manuscript meets style requirements including those for file naming. | - |
| In your Data Availability statement, you have not specified where the minimal data set underlying the results described in your manuscript can be found | We synthesised data from published journal articles. The full reference for each journal article included in this review can be found in the reference list.  Table 1 includes the author and title of all 26 papers included in the review, along with a citation that links to the reference list. | Lines 855-1060.  Table 1 |
| Upon re-submitting your revised manuscript, please upload your study’s minimal underlying data set as either Supporting Information files or to a stable, public repository and include the relevant URLs, DOIs, or accession numbers within your revised cover letter | In the reference list we have included the full references for all 26 journal articles which make up our dataset, along with the DOI where available. | Lines 855-1060. |
| Please upload a copy of Figure 1, to which you refer in your text on page 10. If the figure is no longer to be included as part of the submission please remove all reference to it within the text. | Upon reviewing our original submission we found that **Fig 1. PRISMA diagram reporting the process of identification, screening, and selection of papers for inclusion in the review,** appeared to have been uploaded successfully.  We have spotted some wayward punctuation in our original figure however and will re-upload Fig 1. with these revisions. | -  Fig 1. |
| Please review your reference list to ensure that it is complete and correct. | We have carefully reviewed the reference list and we are confident that it is complete and correct. | Lines 855-1060. |

| Comments from Reviewer 1 | | Location of revisions |
| --- | --- | --- |
| The authors explain the relevance of the review by referring to the increased demands on academics, limited literature on mental health and wellbeing in academia and heterogeneity of the groups working in academia, as well as of the constructs of mental health and wellbeing. These introductory paragraphs are well written and provide a clear understanding to the readers on why such a review is needed.  However, the authors do not explain what they mean by mental health and wellbeing, the potential overlap between the terms or the clear differences. This is attempted in the methods section (lines 137-140); however, these are mostly examples, and the review would benefit from a more thorough definition of the two concepts from the start.  Furthermore, the introduction lacks a clear rationale as of why the authors have chosen to synthesise qualitative published data only. I can see that this is briefly mentioned in the methods section (lines 151-152), but a clear rationale from the beginning would help the readers. | Thank you for this positive feedback.  We have now included a brief paragraph defining the concepts of well-being and mental health.  We have also included a brief paragraph detailing some of the limitations of current quantitative research on this topic and linked this to why we have chosen to focus on synthesizing qualitative work in our systematic review. | -  Lines 69-79  Lines 110-118. |
| Line 141 – I think it would be better to clarify criterion (a) – how did the authors determine if the articles focused sufficiently on researchers’ mental health and well-being experiences? What counted as “sufficient”? Did each article have to investigate both constructs, or was one or the other sufficient for inclusion? | We have amended criterion (d) in the paragraph detailing the inclusion criteria to explicitly state what is meant by ‘sufficiently’ focusing on mental health and well-being. We have clarified that each article needed to focus on either a construct related to mental health or a construct related to well-being (or both) to be included. | Lines 170-174, and line 180. |
| Lines 170-171 – how did the authors decide that these eight papers were the “most immediately relevant and current papers”? | We have clarified what was meant by the eight “most immediately relevant and current papers”. | Lines 210-213. |
| Though the diversity of the research team is a clear strength of the review, and it is great to see that the different backgrounds and expertise of each researcher is discussed, I think it might be an overstatement to say that this ensured that any personal assumptions or blind spots were identified – perhaps this expertise helped to minimise such aspects | Thank you for this positive feedback. We have amended this statement to include the term minimised, rather than ‘challenged and identified’. | Line 232. |
| Results - Well written - Line 394 – I think the authors meant “affecting”? | Thank you for this positive feedback.  We have amended this on line 437. | -  Line 437. |
| Line 646 – While the work here is extremely important and a great start in synthesising researchers’ experiences across a number of geographical regions-  -I think it is important to acknowledge that the representativeness of these experiences is still under question, especially as there were no papers from Africa, one from Asia (only China) and most papers were from developed regions of the world (North America, Europe, Australia and Oceania). Therefore, the authors must be careful when stating that these themes “transcended geographical…boundaries” | Thank you for this positive feedback.  We agree that this statement could be misleading. We have amended this statement to clarify that the seven key themes spanned across the countries, disciplines and career stages mentioned in the included papers, and not generally, as the original statement suggests. | -  Lines 689-690. |

| Comments from Reviewer 2 | | Location of revisions |
| --- | --- | --- |
| This is important work with a potential to better our understanding of the complex relationships between the academic culture, system, work conditions, and scholars’ mental health and well-being. | Thank you for this positive feedback. | - |
| Some working definitions of the main concept in the study should be explicitly stated in the introduction. These are: mental health and wellbeing (plus the difference); early career researchers (ECRs); the difference between “mental health” and “mental health experiences” (does it mean self-reported mental health in comparison to clinical screening tools?). The last is particularly important for those readers who are more familiar with quantitative literature. | We have now included a brief paragraph defining the concepts of well-being and mental health.  We have more clearly defined what we mean by early career researchers in the introduction.  We have also clarified that when discussing mental health and well-being in the context of qualitative data, we are talking about self-report rather than screening tools or measures. | Lines 69-79.  Lines 81-82.  Lines 114-118. |
| I’d suggest using the term “doctoral researchers” more than “doctoral students”. PhD is a training and a job, but it’s very different from studying on the BA or MA levels. The term “doctoral researchers” seems to align better with the effort to mitigate too dominant power dynamics in academia | We have amended our wording from ‘doctoral student’ to ‘doctoral researcher’ throughout the manuscript and in Table 1. (We did not amend quotes or the titles of papers). | Lines 82-818, and Table 1. |
| As the Authors mention, there are some initiatives aiming to incentivise improvements in academia (like Athena SWAN Charter), however, for the sake of completeness, it may be valuable to mention that there are many bottom-up initiatives organised and maintained by ECRs which aim to both describe and improve the situation. It is meaningful that ECRs often take the initiative to help each other while missing enough support from their institutions. It also speaks to the need for peer support, emphasised in the article. I can only make some suggestions from Germany ([http://scholarminds.net](https://eur01.safelinks.protection.outlook.com/?url=http%3A%2F%2Fscholarminds.net%2F&data=04%7C01%7Chelen.nicholls.20%40ucl.ac.uk%7Cc16706ac7ddd488958a308da17e9e461%7C1faf88fea9984c5b93c9210a11d9a5c2%7C0%7C0%7C637848590906545865%7CUnknown%7CTWFpbGZsb3d8eyJWIjoiMC4wLjAwMDAiLCJQIjoiV2luMzIiLCJBTiI6Ik1haWwiLCJXVCI6Mn0%3D%7C3000&sdata=9Jw86G8ayme6iXFM7oMgsXp278itIMh4OAnyn5LLU0w%3D&reserved=0), [https://www.phdnet.mpg.de/n2](https://eur01.safelinks.protection.outlook.com/?url=https%3A%2F%2Fwww.phdnet.mpg.de%2Fn2&data=04%7C01%7Chelen.nicholls.20%40ucl.ac.uk%7Cc16706ac7ddd488958a308da17e9e461%7C1faf88fea9984c5b93c9210a11d9a5c2%7C0%7C0%7C637848590906545865%7CUnknown%7CTWFpbGZsb3d8eyJWIjoiMC4wLjAwMDAiLCJQIjoiV2luMzIiLCJBTiI6Ik1haWwiLCJXVCI6Mn0%3D%7C3000&sdata=q5WjZQCwNk2PRFRrJ0LG8yZdDWCiVIOCTD8tqUimStk%3D&reserved=0)), but I am sure there are more out there in Europe and beyond. | We agree that including mention of ECR created peer support groups would be beneficial and have now included this in the discussion. Thank you for the link to a current ECR peer support group. | Lines 735-739. |
| Figure 2: It’s a very informative figure, but the research question in the middle of the figure doesn’t fit there. Maybe “main themes in mental health/well-being research” or something similar would be more adequate? | Thank you for your positive feedback. We have amended the research question in the middle of the diagram to read as a descriptive title rather than a question – ‘**Researchers’ experiences of working in academia, and the effect these experiences have on their mental health and well-being’.** | Fig 2. |
| Lines 74-79: these two sentences don’t seem to follow one another. Did the Authors mean that secure employment, autonomy, and teamwork are less present in the life of PhD students? | We have clarified here that we are referring to university employees, including academic staff. | Lines 107-108. |
| One thing that is generally not clear is how prevalent the issues are. The Authors should mention the problem with biased data/convenience sample: those who are experiencing mental health/wellbeing issues are more likely to participate in studies investigating these topics, especially when the studies are qualitative (which normally require more time than, for example, forced-choice surveys). One way to discuss this would be to look into recruitment strategies in the analysed papers. Nevertheless, the bias could not be fully excluded that way, so a comment should be made. | We have now included mention of this particular bias (that the views of those who have experienced mental health or well-being related difficulties may be more represented in our analysis) in our ‘strengths and limitations of the meta-synthesis section’.  We have included reference to the fact that those with prior experiences of mental health or well-being related difficulties may be more likely to take part in research exploring these constructs (as suggested), and we have also commented on the fact that some of the included papers specifically sought out participants with symptoms of a mental health difficulty, and others specifically focused on examining more negative constructs which could contribute to poorer mental health and well-being such as stress (which may also contribute to this bias). | Lines 824-831 |
| There is an emphasis on the distinction between mental health and well-being in line 137-140, which is useful given that these two may require different strategies, responsible bodies, time frames, legal structures, and staff to improve the issues. However, this distinction is blurred in the results and neglected in the discussion. If this was done because it is simply hard to point out the differences in the data, it should be mentioned. If the reason was that the 7 identified factors seem to influence both in the same or at least similar way, it is also an interesting comment and should be mentioned. | We have clarified that due to differences in how mental health and well-being were conceptualised and discussed across the included papers, it was then difficult to maintain a distinction between the two concepts when conducting our analysis. | Lines 790-793. |
| Some statements in the text seem a little strong. However probable, these statements may give a false impression of an existing causal relationship which may be an (possibly correct!) interpretation of the Authors (as they admit themselves in the discussion of limitations). If the Authors can elaborate on it further (maybe with more examples), that would be beneficial. If not, perhaps a weaker version should be considered (and maybe suggested for future research to follow up on). Some examples: o In lines 373-374: “Due to this culture of silence however, there was a tendency for researchers to blame any difficulties or dissatisfaction associated with their job on themselves”. However probable, it seems like a rather strong statement between “the culture of silence” and self-blame. o In lines 392-395: “Ultimately, being perceived as meeting or not meeting the expectations (…) can have an impact on a researchers’ confidence in their ability to do their job, not only effecting their well-being, but also their sense of identity as an academic, and thus their sense of belonging to the academic community.” | We agree that the statements presented in the reviewer’s comment are a little strong. We have amended both statements to lessen the link between ‘cause’ and ‘effect’. | Line 416-419, and line 436. |
| Just a comment: I applaud the transparency achieved with the “Reflexivity” part of the manuscript and the discussion of researchers’ influence in “Strengths and limitations of the included papers”. | Thank you for this positive feedback. | - |
| There is a growing number of quantitative works using screening tools and custom questionnaires to provide evidence for elevated levels of depression, anxiety, burnout, etc. in ECRs. Even though this is a qualitative analysis, the authors insist on scarcity of research done in the field of mental health of academics without citing many of works that have directly estimated the scope of the problem. If only for the sake of dissemination and relatedness between studies using different methods but on the same topic, it should be at least noted that they exist. To name a few: o Evans, T. M., Bira, L., Gastelum, J. B., Weiss, L. T., & Vanderford, N. L. (2018). Evidence for a mental health crisis in graduate education. Nature Biotechnology, 36 (3), 282–284. [https://doi.org/10.1038/nbt.4089](https://eur01.safelinks.protection.outlook.com/?url=https%3A%2F%2Fdoi.org%2F10.1038%2Fnbt.4089&data=04%7C01%7Chelen.nicholls.20%40ucl.ac.uk%7Cc16706ac7ddd488958a308da17e9e461%7C1faf88fea9984c5b93c9210a11d9a5c2%7C0%7C0%7C637848590906545865%7CUnknown%7CTWFpbGZsb3d8eyJWIjoiMC4wLjAwMDAiLCJQIjoiV2luMzIiLCJBTiI6Ik1haWwiLCJXVCI6Mn0%3D%7C3000&sdata=iZNadyfVoy7i4O1pkGNgn9Z%2FCQCxx%2FyZxXy6gcAlcXI%3D&reserved=0) o Satinsky, E. N., Kimura, T., Kiang, M. V., Abebe, R., Cunningham, S., Lee, H., ... Tsai, A. C. (2021). Systematic review and meta-analysis of depression, anxiety, and suicidal ideation among Ph.D. students. Scientific Reports, 11 (1), 1–12. [https://doi.org/10.1038/s41598-021-93687-7](https://eur01.safelinks.protection.outlook.com/?url=https%3A%2F%2Fdoi.org%2F10.1038%2Fs41598-021-93687-7&data=04%7C01%7Chelen.nicholls.20%40ucl.ac.uk%7Cc16706ac7ddd488958a308da17e9e461%7C1faf88fea9984c5b93c9210a11d9a5c2%7C0%7C0%7C637848590906545865%7CUnknown%7CTWFpbGZsb3d8eyJWIjoiMC4wLjAwMDAiLCJQIjoiV2luMzIiLCJBTiI6Ik1haWwiLCJXVCI6Mn0%3D%7C3000&sdata=KgRTxxzHPMtKCxKkc0PFfG1SyZugDSuBwIxxDzyaVdU%3D&reserved=0) o Woolston, C. (2017). Graduate survey: A love–hurt relationship. Nature, 550 (7677), 549–552. [https://doi.org/10.1038/nj7677-549a](https://eur01.safelinks.protection.outlook.com/?url=https%3A%2F%2Fdoi.org%2F10.1038%2Fnj7677-549a&data=04%7C01%7Chelen.nicholls.20%40ucl.ac.uk%7Cc16706ac7ddd488958a308da17e9e461%7C1faf88fea9984c5b93c9210a11d9a5c2%7C0%7C0%7C637848590906545865%7CUnknown%7CTWFpbGZsb3d8eyJWIjoiMC4wLjAwMDAiLCJQIjoiV2luMzIiLCJBTiI6Ik1haWwiLCJXVCI6Mn0%3D%7C3000&sdata=CbnaBxI8R4lO%2Bq4CPprK6PsYND15v0bJ97Jcqk5DuBY%3D&reserved=0) | Thank you for the provided references. In the introduction, we have included a brief paragraph detailing some of the quantitative literature available on this topic which highlights the prevalence and severity of mental ill health among academic researchers. | Lines 86-105. |
| After all the work the Authors put into this analysis, and given their insightful conclusions, as a reader I remain wanting to learn more about their own suggestions for how to improve the situation in academia. They appear here and there in the discussion, yet, even if only speculative and brief, the Authors could use their acquired insights in the topic to clearly name some evident candidates for a change. However, this is a personal choice of the Authors and leaving the several suggestions only embedded in the discussion would not make the paper lacking. | Thank you for this positive feedback. After careful consideration, we have chosen to leave the original suggestions for change embedded in the discussion. We have chosen to do this as whilst all of the included papers provided valuable insight into the mental health and well-being of academic researchers, the differences in how the terms mental health and well-being were conceptualised make it difficult to draw out clear and explicit ways in which researchers’ mental health and/or well-being could be more effectively supported. | - |
| Two tiny things unrelated to the content: There’s a typo in line 93 (aide instead of aid) and there are some misplaced or missing quotation marks, apostrophes, and brackets throughout the text. | We have amended the typo, and carefully scrolled through the manuscript to ensure that the punctuation is now correct. | Lines 19-850. |

Thank you again for considering our paper. We hope these amendments address the points raised and very much look forward to hearing further from you.

With kind regards,

Helen Nicholls

Doctoral student

UCL Division of Psychiatry
