# Supplementary material for: The impact of working in academia on researchers’ mental health and well-being: A systematic review and qualitative meta-synthesis
Source: PLoS One. 2022 May 25;17(5):e0268890. doi: 10.1371/journal.pone.0268890 (PMC9132292; doi:10.1371/journal.pone.0268890)
Supplement: Attachment — Submitted filename: Response to Reviewers.docx [file pone.0268890.s004.docx]

**UCL Division of Psychiatry**

Helen Nicholls

UCL Division of Psychiatry

149 Tottenham Court Road

London, W1T 7NF, UK

helen.nicholls.20@ucl.ac.uk

Giuseppe Filiberto Serraino,

Academic Editor,

PLOS ONE

5^th^ May, 2022

Dear Dr Giuseppe Filiberto Serraino,

We would like to thank both you and the reviewers for reviewing our manuscript entitled: The impact of working in academia on researchers' mental health and well-being: A systematic review and qualitative meta-synthesis (PONE-D-22-03203R1).

We have provided a point-by-point response to the comments made below.

| Journal requirements | | Location of revisions |
| --- | --- | --- |
| Please review your reference list to ensure that it is complete and correct. If you have cited papers that have been retracted, please include the rationale for doing so in the manuscript text, or remove these references and replace them with relevant current references. Any changes to the reference list should be mentioned in the rebuttal letter that accompanies your revised manuscript. If you need to cite a retracted article, indicate the article’s retracted status in the References list and also include a citation and full reference for the retraction notice. | Thank you for your comment. We have gone through the reference list to ensure that all references have a doi and/or volume and page numbers. For references that are not journal articles, we have provided a url/link to where the text can be sourced. We have also employed a ‘Plos One’ citation style that was accessed through our reference management software – Zotero.  The following original reference,  ‘Moran H, Karlin L, Lauchlan E, Rappaport SJ, Bleasdale B, Wild L, et al. Understanding Research Culture: What researchers think about the culture they work in. Wellcome Open Research. 2020;5: 201’,  has been replaced with:  ‘Wellcome. What researchers think about the culture they work in. 15 Jan 2020. Available: <https://wellcome.org/sites/default/files/what-researchers-think-about-the-culture-they-work-in.pdf>’  We replaced the original reference to provide a direct link to the Wellcome report that was accessed.  The following original reference,  ‘Rosser SV. Academic women in STEM faculty: Views beyond a decade after POWRE. Academic women in STEM faculty: Views beyond a decade after POWRE. 2017. doi:10.1007/978-3-319-48793-9’  has been replaced with:  ‘Rosser SV, Barnard S, Carnes M, Munir F. Athena SWAN and ADVANCE: effectiveness and lessons learned. The Lancet. 2019;393: 604–608. doi:10.1016/S0140-6736(18)33213-6’  We felt the new reference more fully reflected our point about the limitations of initiatives aimed at addressing inequality in academia. | Lines 854-1071  Lines 876-878  Lines 1066-1068 |

| Comments from Reviewer 2 | | Location of revisions |
| --- | --- | --- |
| Thank you for your revisions. I believe this is important work which should be shared with other researchers. A tiny comment - while reading the revised version, I came across a typo in line 93: poor work like balance (instead of life). Congratulations for the authors on the work and good luck for future projects. | Thank you for this positive feedback and thank you for spotting the typo – we have now amended this. | Line 92 |

Thank you again for considering our paper. We hope these amendments address the points raised and very much look forward to hearing further from you.

With kind regards,

Helen Nicholls

Doctoral student

UCL Division of Psychiatry
